# Next-Generation Cancer Immunotherapy Targeting Glypican-3

**DOI:** 10.3389/fonc.2019.00248

**Published:** 2019-04-10

**Authors:** Yasuhiro Shimizu, Toshihiro Suzuki, Toshiaki Yoshikawa, Itaru Endo, Tetsuya Nakatsura

**Affiliations:** ^1^Division of Cancer Immunotherapy, Exploratory Oncology Research and Clinical Trial Center, National Cancer Center, Kashiwa, Japan; ^2^Department of Gastroenterological Surgery, Yokohama City University Graduate School of Medicine, Yokohama, Japan

**Keywords:** glypican-3 (GPC3), cancer antigen, cancer immunotherapy, cancer vaccine, cytotoxic T cell, TCR-engineered T cell therapy, CAR-T therapy

## Abstract

Glypican-3 (GPC3), a 65 kD protein consisting of 580 amino acids, is a heparan sulfate proteoglycan bound to the cell membrane by glycosylphosphatidylinositol. This protein is expressed in the liver and the kidney of healthy fetuses but is hardly expressed in adults, except in the placenta. Contrarily, GPC3 is specifically expressed in hepatocellular carcinoma (HCC), ovarian clear cell carcinoma, melanoma, squamous cell carcinoma of the lung, hepatoblastoma, nephroblastoma (Wilms tumor), yolk sac tumor, and some pediatric cancers. Although the precise function of GPC3 remains unclear, it has been strongly suggested that it is related to the malignant transformation of HCC. We identified GPC3 as a promising target for cancer immunotherapy and have been working on the development of cancer immunotherapeutic agents targeting it through clinical trials. In some trials, it was revealed that the GPC3 peptide vaccines we developed using human leukocyte antigen-A24- and A2-restricted GPC3-derived peptides could induce GPC3-specific cytotoxic T cells in most vaccinated patients and thereby improve their prognosis. To further improve the clinical efficacy of cancer immunotherapy targeting GPC3, we are also developing next-generation therapeutic strategies using T cells engineered to express antigen-specific T-cell receptor or chimeric antigen receptor. In addition, we have successfully monitored the levels of serum full-length GPC3 protein, which is somehow secreted in the blood. The utility of GPC3 as a biomarker for predicting tumor recurrence and treatment efficacy is now being considered. In this review article, we summarize the results of clinical trials carried out by our team and describe the novel agent targeting the cancer-specific shared antigen, GPC3.

## Introduction

Therapeutic approaches that exploit the immune system are a promising alternative strategy to surgery, radiotherapy, and anticancer drug therapy for cancer treatment. Recent studies have shown that immune checkpoint inhibiters (ICIs) such as antibodies against CTLA-4, programmed cell death (PD)-1, and programmed death ligand 1 have potent and long-term antitumor effects (1, 2); in 2018, Tasuku Honjo and James P Alison won the Nobel Prize for Medicine for their contribution to their development. The extremely high response rates to chimeric antigen receptor-introduced T cell therapy (CAR-T therapy) for cluster of differentiation (CD)19^+^ hematopoietic tumors and tumor-infiltrative T cell transfer therapy for malignant melanoma have provided further evidence for the efficacy of cancer immunotherapies (3, 4). Additionally, tumor-specific mutant antigens (neoantigen) have attracted attention for their therapeutic potential and clinical trials of personalized cancer vaccines that target neoantigens have been initiated in Europe and the United States (5–7) Meanwhile, peptide vaccines against shared antigens have been developed in Japan but have not yet been approved by the Pharmaceutical Affairs Law.

With the exception of Hodgkin's lymphoma, response rates to ICIs are estimated as no more than 30% in the case of melanoma and 10–20% for other cancers (8, 9). An outstanding challenge is to develop effective therapies for patients who are unresponsive to ICIs. To address this issue, two points must be considered: firstly, the extents to which cancer-responsive T cells are active in patients' bodies; and secondly, how their infiltration into tumors can be enhanced. In cases where cancer-specific effector T cell counts are low, their numbers must be increased by T cell transfer therapy or else the T cells must be induced with cancer vaccines. Inducing inflammation at tumor loci by administration of adjuvants or by chemo- or radiation therapy has been shown to promote T cell infiltration into tumors (10), and ICIs and individualized cancer vaccines derived from neoantigens are thought to be effective in patients with a high frequency of somatic mutations (11). On the other hand, it is difficult to enhance anti-cancer immune responses and even if immunosuppression is overcome (12) in patients with a low, making it necessary to target not only neoantigens but also shared antigens like glypican (GPC)-3. Our clinical trials have shown that cancer peptide vaccines targeting shared antigens can induce peptide-specific cytotoxic T lymphocytes (CTLs) in vaccinated patients without eliciting non-specific autoimmune responses. In addition, gene-based therapy using T cell receptor (TCR) obtained from CTL clones induced by cancer peptide vaccine and isolated from vaccinated patients is expected to have more potent antitumor effects (13). In this review, we summarize the results of cancer immunotherapy targeting GPC3 based on our experience and outline the future prospects.

### GPC3

**Figure 1:**
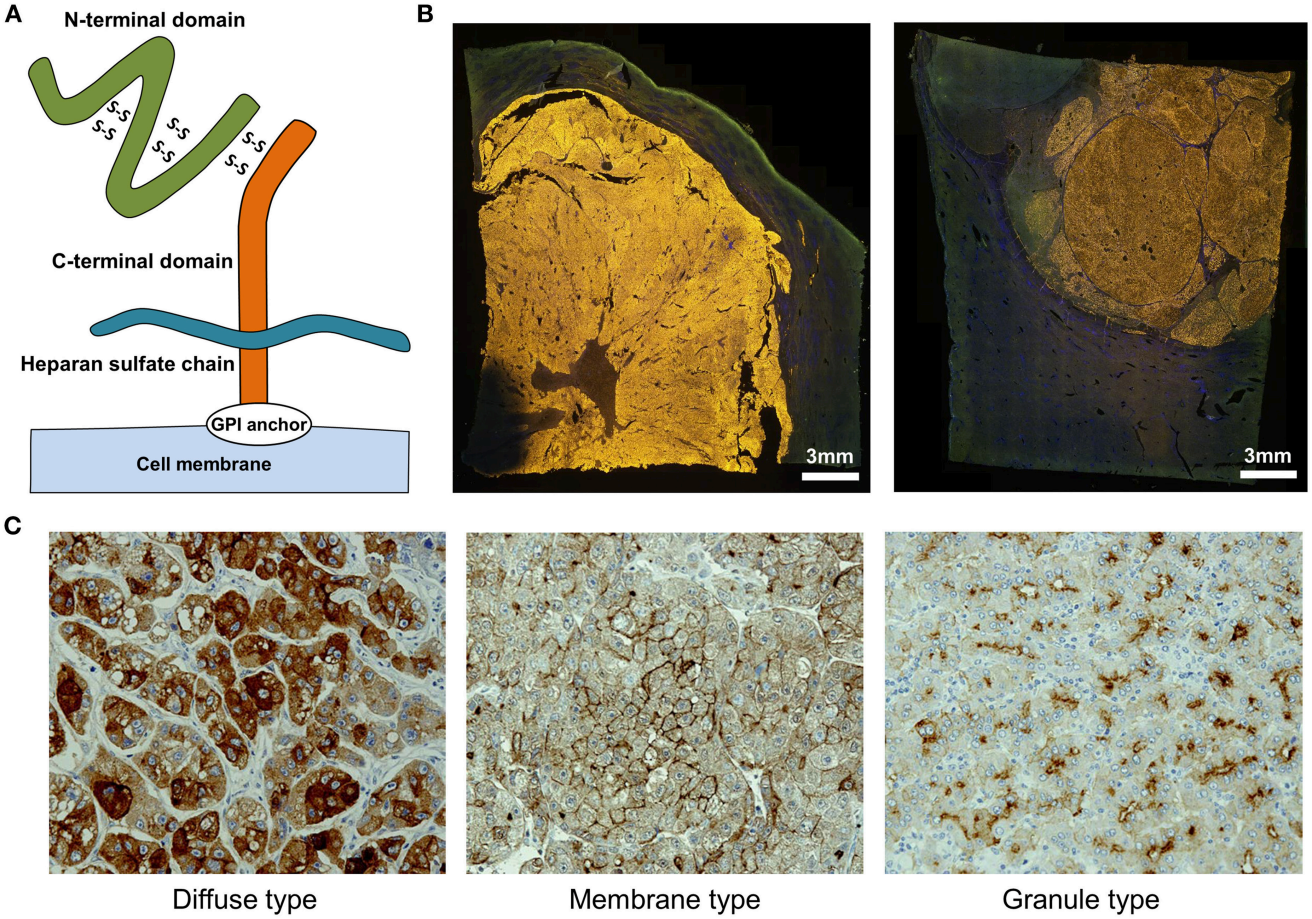
Characteristics of GPC3. **(A)** Schema of GPC3. **(B)** Fluorescence micrographs of GPC3-positive HCC. GPC3 labeling appears as a yellow color. **(C)** GPC3 expression patterns can be classified into diffuse, membrane, and granule types by immunohistochemistry.

We identified GPC3 in a cDNA microarray screen of several tens of thousands of genes for novel cancer antigens (14). GPC3, 65-kDa protein consisting of 580 amino acids is a heparan sulfate chain proteoglycan bound to the cell membrane by a glycosylphosphatidylinositol (GPI) anchor (Figure 1A) (15). GPC3 regulates cell proliferation signals by binding growth factors such as Wnt, fibroblast growth factor, and insulin-like growth factor and plays an important role in the proliferation and differentiation of embryonic cells (16–18). In addition, the gene is present on the X chromosome (Xq26) and shows high homology between humans and mice. Gene mutations and deletions cause gigantism with various malformations and Simpson-Golabi-Behmel syndrome in humans, with similar phenotypic manifestations in mice (16, 19). GPC3 is expressed in various fetal tissues (liver, lung, kidney, and placenta) but is not detected in normal postnatal tissue due to DNA methylation-induced epigenetic silencing (20, 21). On the other hand, GPC3 is expressed in hepatocellular carcinoma (HCC), melanomas, ovarian clear cell carcinoma (OCCC), lung squamous cell carcinomas, and some childhood cancers (hepatoblastomas, nephroblastomas, and yolk sac tumors) (14, 21). Particularly, GPC3 is detected in ≥80% of patients with HCC caused by hepatitis B or C (Figure 1B) (14, 22, 23). The function of membrane-anchored GPC3 in these cancers is unknown, but it is likely involved in the neoplastic transformation of HCC (23). Additionally, immunohistochemical analysis of HCC tissues have revealed at least three GPC3 expression patterns, which we have classified as diffuse, membrane-localized, and granular (Figure 1C) (Shimizu, manuscript in preparation).

Membrane-bound GPC3 can be cleaved and secreted into the blood (Figure 1A). Mammalian GPC family members are cleaved at the GPI anchor level by endogenous GPI phospholipase D (24). It was previously proposed that Notum (a conserved secretory feedback inhibitory protein of the Wnt signaling pathway) targets the GPI anchor in a manner similar to phospholipase and draws the GPC/Wnt complex away from the cell surface to inhibit Wnt signaling; however, it is now thought to control the signal without acting on the anchor (25, 26). It is presumed that GPC3 is cleaved between Arg358 and Ser359, which releases the N-terminal region as a soluble protein from cancer cells into the circulation (27). Thus, various forms of GPC3 protein are present in blood, although their functions remain unclear.

Given these features, GPC3 is an ideal target for cancer immunotherapy. We identified each peptide that can bind to human leukocyte antigen (HLA)-A24 or -A2 and induce GPC3 peptide-specific CTLs (28, 29). Furthermore, we conducted clinical trials of vaccines based on these peptides (30–34). In the future, we envision an array of GPC3-based strategies, not only as cancer vaccines, but also in antibody therapy, adoptive immunotherapy with TCR- or CAR-transduced T cells, and others. We also anticipate that plasma GPC3 will be validated as a biomarker for HCC or for evaluating the efficacy of cancer immunotherapy against GPC3.

### Preclinical Studies of GPC3-Derived Peptide Vaccine

We have identified HLA-A24- and HLA-A2-restricted GPC3-derived peptides; approximately 60% of Japanese are positive for HLA-A24 and 40% for HLA-A2, which is the major type in Europeans and North Americans (28, 29). Peptide-specific CTLs were induced in mice immunized with these GPC3 peptides, which exerted antitumor effects without eliciting an autoimmune response (28, 29). In preparation for clinical trials using these HLA-restricted peptides, we investigated whether differences in peptide dosage would affect the efficacy of vaccination, and performed studies in mice to determine the optimal adjuvant (35). We compared five groups: peptide-only and peptide combined with incomplete Freund's adjuvant (IFA), CpG, α-GalCel, or aluminum and found that the GPC3-specific CTLs were only induced in the IFA combination group, demonstrating that the peptide alone was ineffective. We therefore used the peptides plus IFA as cancer vaccines in clinical trials and observed that stronger immune responses were induced by varying peptide dosage.

### Phase I Clinical Trial of GPC3 Peptide Vaccines Against Advanced HCC

At the National Cancer Center Hospital East (Kashiwa, Japan), we conducted a phase I clinical trial of GPC3 peptide vaccines in a cohort comprising 33 cases of advanced HCC from February 2007 to November 2009 (UMIN Clinical Trials Registry: 000001395) (Table 1) (30, 38). The primary endpoints were safety and immune response. In a dose escalation study of 0.3, 1, 3, 10, and 30 mg there was no dose-limiting toxicity (DLT), making it difficult to determine the maximum tolerated dose. Although the partial clinical response in a patient treated with 30 mg as well as dose-dependent immunological reactions suggested a greater efficacy of high dosages, a dose of 30 mg required a total vaccine volume of 6 ml, which was difficult to administer and caused pain along with reddening and induration at the site of administration. Based on these observations, we determined that a dosage of 3 mg would be appropriate for the next-phase trial.

**Table 1:** Summary of clinical trials for cancer immunotherapy targeting GPC3.

**Trial**	**ID**	**References**	**Key inclusion criteria**	**Primary endpoint**	**Results**
**OUR CLINICAL TRIALS**					
Phase I clinical study of GPC3 peptide vaccine in patients with advanced HCC	UMIN 000001395	Sawada et al. (30)	Advanced HCC patient	(1) Adverse effects of GPC3 vaccine	GPC3 vaccination was well-tolerated; the vaccine induced a GPC3-specific CTL response in 30/33 patients (91%)
				(2) GPC3-specific immune responses to GPC3 vaccine	
Clinical study evaluating immunological efficacy of GPC3 peptide vaccine in patients with advanced HCC	UMIN 000005093	Tsuchiya et al. (34)	Advanced HCC patient	Increased percentage of GPC3 peptide-specific CD8-positive T lymphocytes in blood and tumor	After vaccination, the number of GPC3 peptide-specific CTLs in PBMC was increased in 9 of 11 patients; tumor biopsy specimens obtained from three patients post-vaccination revealed CTL infiltration
Phase II study of GPC3 peptide vaccine as adjuvant treatment for HCC after surgical resection or RFA	UMIN 000002614	Sawada et al. (31)	(1) Diagnosed as initial HCC	1- and 2-year recurrence rate	1- and 2-year recurrence rates were 24.4 and 53.7%, respectively; the primary endpoint was not reached
			(2) Subjects who underwent potentially curative surgical resection or RFA for treatment of HCC		
Phase II study of GPC3 peptide vaccine for treatment of OCCC	UMIN 000003696	Suzuki et al. (32)	Advanced OCCC patient	DCR at 6 months	DCR at 6 months was 9.4% (3/32)
Phase I study of GPC3 peptide vaccine for pediatric patients with refractory tumors	UMIN 000006357	Tsuchiya et al. (33)	(1) Patients with refractory, recurrent, or progressive status (progressive group)	Incidence of DLT	No DLT or dose-specific adverse events were observed
			(2) Patients in remission without chance of cure (remission group)		
			(3) Patients in partial remission or with stable disease (partial remission group)		
**OTHER CLINICAL TRIALS**					
First-in-man phase I study of GC33, a novel recombinant humanized antibody against glypican-3, in patients with advanced hepatocellular carcinoma	NCT 00746317	Zhu et al. (36)	Patients with measurable, histologically demonstrated advanced HCC	Maximum tolerated dose was not reached as there was no DLT up to the highest planned dose level. Median TTP was 26.0 and 7.1 weeks in the high and low GPC3 expression groups, respectively	
Japanese phase I study of GC33, a humanized antibody against glypican-3 for advanced hepatocellular carcinoma	JapicCTI 101255	Ikeda et al. (37)	Japanese patients with advanced HCC	No DLT observed in any patient up to the highest planned dose; 7/13 patients showed SD, 6/13 showed PD, and 3/13 showed long-term SD >5 months	

*CTL, cytotoxic T lymphocyte; DCR, disease control rate; DLT, dose-limiting toxicity; GPC3, glypican-3; HCC, hepatocellular carcinoma; OCCC, ovarian clear cell carcinoma; PBMC, peripheral blood mononuclear cell; PD, progressive disease; RFA, radiofrequency ablation; SD, stable disease; TTP, time to progression*.

In this first-in-human study of GPC3 peptide vaccines, we confirmed the safety of GPC3 peptide vaccines and obtained promising clinical results. We also detected an elevation in peptide-specific CTL counts in peripheral blood by interferon-γ enzyme-linked immunosorbent spot assay, demonstrating that these vaccines can induce immune responses even in humans. By analyzing tumor biopsies we identified cases where infiltrating CD8^+^ CTLs were present after but not before vaccination, confirming the immunological effects of our vaccines (30, 38).

A subsequent phase I trial was initiated to investigate the extent of CTL infiltration into tumor tissues (UMIN Clinical Trials Registry: 000005093) by analyzing tumor biopsies obtained before and after vaccination (Table 1) (33). The primary endpoint was GPC3 peptide-specific immune response induced by vaccination. However, this trial was conducted after approval of sorafenib treatment, and patients with extremely late-stage HCC were registered only after sorafenib had ceased to be effective; indeed, most of the patients showed negligible response to the vaccine, probably because of their endogenous immunosuppressive states. Additionally, post-vaccination biopsies were completed in just 11 cases. Nevertheless, we were able to glean useful data from this trial: in one case the HCC tissue became inflamed and then necrotic after two injections of the vaccine, despite ongoing liver dysfunction (39); and we established GPC3 peptide-specific CTL clones from a tumor biopsy specimen (33).

### Phase II Clinical Trial to Investigate Relapse Prevention Following Radical Treatment of HCC

We performed a single-arm phase II clinical trial to evaluate 1- and 2-year relapse rates in 41 cases following radical treatment of HCC using GPC3 peptide vaccine as adjuvant therapy (Table 1) (31). GPC3 peptide-specific CTL responses were detected in 35 of the 41 patients (85.4%) after vaccination. Since the absence of GPC3 expression is correlated with good prognosis, we compared patients with GPC3-positive HCC and control subjects and found that post-surgical administration of GPC3 peptide vaccine can extend the recurrence-free survival period; moreover, overall survival was prolonged in cases where CTL induction was observed. This is likely due to the suppression of GPC3-positive HCC with highly malignant features. We plan to report these results in the future (Miura, manuscript in preparation). Meanwhile, there were two cases of relapse despite the presence of numerous induced peptide-specific CTLs in peripheral blood due to vaccine administration. In these cases, GPC3 was expressed in the primary tumor, but in recurrent cancer, the expression was undetectable (31). These observations suggest that peptide vaccine targeting one type of shared antigen—which could eliminate tumor cells expressing the antigen—may not completely prevent tumor growth due to the increased heterogeneity of the cancer. In such instances cancer peptide vaccines that target multiple shared antigens or neoantigens may be more effective. Furthermore, in nine patients whose recurrent tumors expressed GPC3, the frequencies of GPC3-specific CTLs tended to be lower than those in the aforementioned two patients. Although peptide-specific CTLs were induced by vaccination, the reduction of GPC3-positive HCC recurrence due to the peptide vaccine might depend on the strength of CTL induction. We also considered identifying helper T (Th) cell epitopes in the vaccinated patients (40). Our study revealed that GPC3-derived long peptides-specific and HLA class II-restricted CD4± T-cell responses were observed in 14 of 20 patients, and the presence of the specific Th cells was correlated with prolonged overall survival (40). We expect clinical trials to confirm the recurrence prevention effects of peptide vaccines after resection in patients with GPC3-positive HCC.

### Clinical Trials for Advanced OCCC

We also observed the antitumor effects of GPC3 peptide vaccine in advanced OCCC in clinical trials performed at Nagoya University (UMIN Clinical Trials Registry: 000003696) (Table 1) (32, 41). The primary endpoint was disease control rate (DCR) at 6 months. Two cases showed partial response (PR) and one showed stable disease (SD); DCR at 6 months was 9.4% (3/32 cases). While response rates tended to be higher than for HCC, this may be due to differences in tumor quantity. OCCC is extremely difficult to cure with existing anticancer drugs, lending urgency to the development of effective cancer immunotherapies.

### Clinical Trials for Refractory Pediatric Cancer

As mentioned above, GPC3 is expressed in some pediatric cancers, including hepatoblastoma, nephroblastoma (Wilms' tumor), and yolk sac tumors. We performed a multicenter clinical trial that included GPC3-positive refractory pediatric cancer cases (UMIN Clinical Trials Registry: 000006357) (Table 1) (34); 18 patients received GPC3 peptide vaccination. DLT—the primary endpoint—was not observed, and the vaccine induced a GPC3-specific CTL response in 7/18 patients (39%), nearly all of whom belonged to the remission group and were hepatoblastoma patients. In contrast, GPC3-specific CTL frequency was not increased in the refractory advanced progression group. These results suggest that the vaccine can prevent recurrence of hepatoblastoma after the second remission, a period in which relapse is generally considered as unavoidable. Furthermore, recurrence-free survival of more than 4 years was observed in all five patients with hepatoblastoma.

### Intratumoral Vaccination Therapy

Tumor cells reduce their antigen presentation to escape host immune surveillance mechanisms (42), which is a major challenge in the development of effective cancer vaccines. HLA class I expression is reduced or absent in 16–50% of various malignancies (43). To circumvent this problem, we developed an intratumoral injection method for peptide vaccines that has been tested in mice. This mode of delivery enhanced anti-tumor activity as compared to conventional subcutaneous injection and induced systemic immune responses that inhibited the growth of metastasized tumors (44). Moreover, combining intratumoral peptide vaccine injection and anti-PD-1 blocking antibody could elicit enhanced antitumor effects by inducing the upregulation of PD-1 on the surface of CTLs (45–47). This may be applicable not only to primary tumors but also to distant metastatic sites, which could be targeted by loading peptide into HLA class I of tumor cells.

### Therapy With GPC3 Peptide-Specific CTL Clones Established From Vaccinated Patients

In our clinical trials of GPC3 peptide vaccine, we successfully established multiple types of GPC3 peptide-specific CTL clones derived from the peripheral blood and cancer tissue of vaccinated patients (34, 38, 48). Some of these clones have a strong ability to kill cancer cells presenting GPC3 peptide *in vitro*. By cloning these TCRs, we are currently developing an adoptive immunotherapy approach based on these TCR-transduced T cells. GPC3 peptide-specific TCRs were cloned from CTLs obtained from patients who showed no adverse reactions to the peptide vaccine other than reddening and local swelling at the administration site, thus guaranteeing vaccine safety. Adoptive immunotherapy with TCR-transduced T cells is generally considered as having superior antitumor effects to peptide vaccine therapy, and their application to advanced cancers presenting GPC3 peptide is highly anticipated.

### Antibody Therapy Targeting Membrane GPC3

GC33, a humanized monoclonal antibody against GPC3, has been shown to induce antibody-dependent cell-mediated cytotoxicity against GPC3-positive HCC cell lines and elicit anti-tumor effects in patient-derived xenograft cancer models (49). In a first-in-human phase I trial performed in the United States for patients with advanced HCC, GC33 was well-tolerated and antitumor effects were observed in some patients with high GPC3 expression HCC (Table 1) (36). A phase I trial of GC33 carried out in Japan confirmed its tolerability. While there were no complete response or PR cases, SD was achieved by 7 of 13 patients who received the treatment, three of whom maintained this status for 3 or more months (Table 1) (37). A randomized, placebo-controlled, double-blind, multicenter phase II trial of GC33 is presently underway (Table 2). In addition, the results of a basic research study on ERY974, an anti- GPC3/CD3 bi-specific T cell-redirecting antibody were recently reported (50). A phase I clinical trial to confirm its toxicity is currently underway in which there is no limitation to the cancer type if the primary tumor is GPC3-positive (Table 2).

**Table 2:** Summary of clinical trials for cancer immunotherapy targeting GPC3.

**Trial**	**Patients**	**ID**
A randomized, placebo-controlled, double-blind, multicenter phase II trial of intravenous GC33 at 1,600 mg Q2W in previously treated patients with unresectable advanced or metastatic HCC	Histologically confirmed HCC	NCT 01507168
A phase I dose escalation and cohort expansion study of ERY974, an anti-GPC3/CD3 bispecific antibody, in patients with advanced solid tumors	Patients with GPC3-positive advanced solid tumors not amenable to standard therapy or for which standard therapy was not available or not indicated	NCT 02748837
A phase I study of anti-GPC3 chimeric antigen receptor modified T cells in Chinese patients with refractory or relapsed GPC3 + HCC	Patients with GPC3-positive HCC	NCT 02395250
Glypican 3-specific chimeric antigen receptor expressing T cells as immunotherapy for patients with HCC	Patients with GPC3-positive HCC	NCT 02905188

*GPC3, glypican-3; HCC, hepatocellular carcinoma*.

### Development of CAR-T Therapy Targeting Membrane GPC3

While CAR-T therapy has shown a remarkable response rate exceeding 80% against B cell blood tumors (51, 52), fully promising results have not yet been obtained against solid carcinomas, such as glioblastoma (53), pancreatic cancer (54), and prostate cancer (55, 56). These insufficient responses were thought to be caused by tumor heterogeneity, immunosuppressive mechanism in the tumor microenvironment, and insufficient accumulation of CAR-T cells in the tumor (57). One option for ameliorating these problems is IL-7/CCL19 expressing CAR-T cells (58). These cells promoted the migration and activation of not only CAR-T cells but also T cells and dendritic cells at the tumor locus, thereby demonstrating strong anti-tumor effect on solid tumors in mice (58). Moreover, in order to avoid on-target off-tumor toxicity, it is necessary to select an excellent cancer antigen with high tumor specificity. In this regard, GPC3 is considered an ideal target as described above. The development of CAR-T therapy targeting solid tumors is underway around the world, and therapies based on GPC3 antibody gene (GPC3-CAR) have been developed (59, 60). In China and United States, clinical trials of GPC3-CAR therapy against GPC3-positive HCC have already begun (Table 2). By combining it with new technologies that supplement the weakness of CAR-T therapy, it is desired that the clinical effect against solid tumors would be further improved.

### Development of a Novel Immunotherapy Using Induced Pluripotent Stem (iPS) Cells

We think that there is significant value to using T cells derived from iPS cells for the following three reasons: (i) they eliminate the effects of effector T cell exhaustion and aging; (ii) they enable reliable gene manipulation at the iPS cell stage; and (iii) they make it possible to continue the treatment over a long period of time. By using an iPS cell bank, we may be able to develop innovative adoptive immunotherapeutics that can be universally adopted at a low cost. In addition, since some patients may have T cells that lack intrinsic anti-tumor potency, an alternative, third party off the shelf T cell product might be a good alternative. Kaneko et al. at Kyoto University in Japan have successfully prevented unwanted reconstruction of TCRs by excluding genes causing TCR remodeling in T cells derived from iPS cells through genome editing, and showed that cytotoxic T cells with high avidity for cancer cells can be induced through this process (61). We are currently developing an iPS cell-derived TCR-T cell (TCR-iPS-T cell) strategy for expressing GPC3-peptide specific TCR in collaboration with their team. In detail, the cell was created by transducing HLA haplotype homologous iPS cell clones with HLA-A^*^24:02 restricted GPC3_298−306_ peptide-specific TCR gene using a lentiviral vector and then stimulating differentiation (61). The findings to date indicate that these TCR-iPS-T cells derived from an HLA homozygous iPS cell stock showed both cancer antigen-specific cytotoxic effects caused by CAR and non-specific cytotoxicity due to stimulation of natural killer cell ligands against GPC3-positive cell lines. These results may lead to the development of a novel type of immunotherapy that can prevent the suppression of anti-cancer effects caused by immune editing against a particular antigen.

### Biomarkers Based on Serum Full-Length GPC3

As described above, GPC3 is released into the serum of HCC patients, and its utility as a tumor marker has been reported (62, 63). We established a novel sandwich enzyme-linked immunosorbent assay system for predicting HCC recurrence after surgery based on post-operative elevation of serum GPC3 level (64). In immunohistochemical analyses of GPC3-positive HCC specimens, a few surrounding normal cells also weakly expressed GPC3, which we believe contribute to post-operative GPC3 secretion and recurrence (64). In partnership with a private company, we have developed an assay to quantify serum full-length GPC3 level, which is the most physiologically relevant parameter. In combination with existing tumor markers such as alpha-fetoprotein and protein induced by vitamin K absence II, we successfully predicted early recurrence of HCC after surgery (Miura, manuscript in preparation). We are presently examining whether this assay can predict hepatocellular carcinogenesis in patients with chronic hepatitis and cirrhosis as well as response to anti-GPC3 therapy.

### Companion Diagnosis for Cancer Immunotherapy Against GPC3

**Figure 2:**
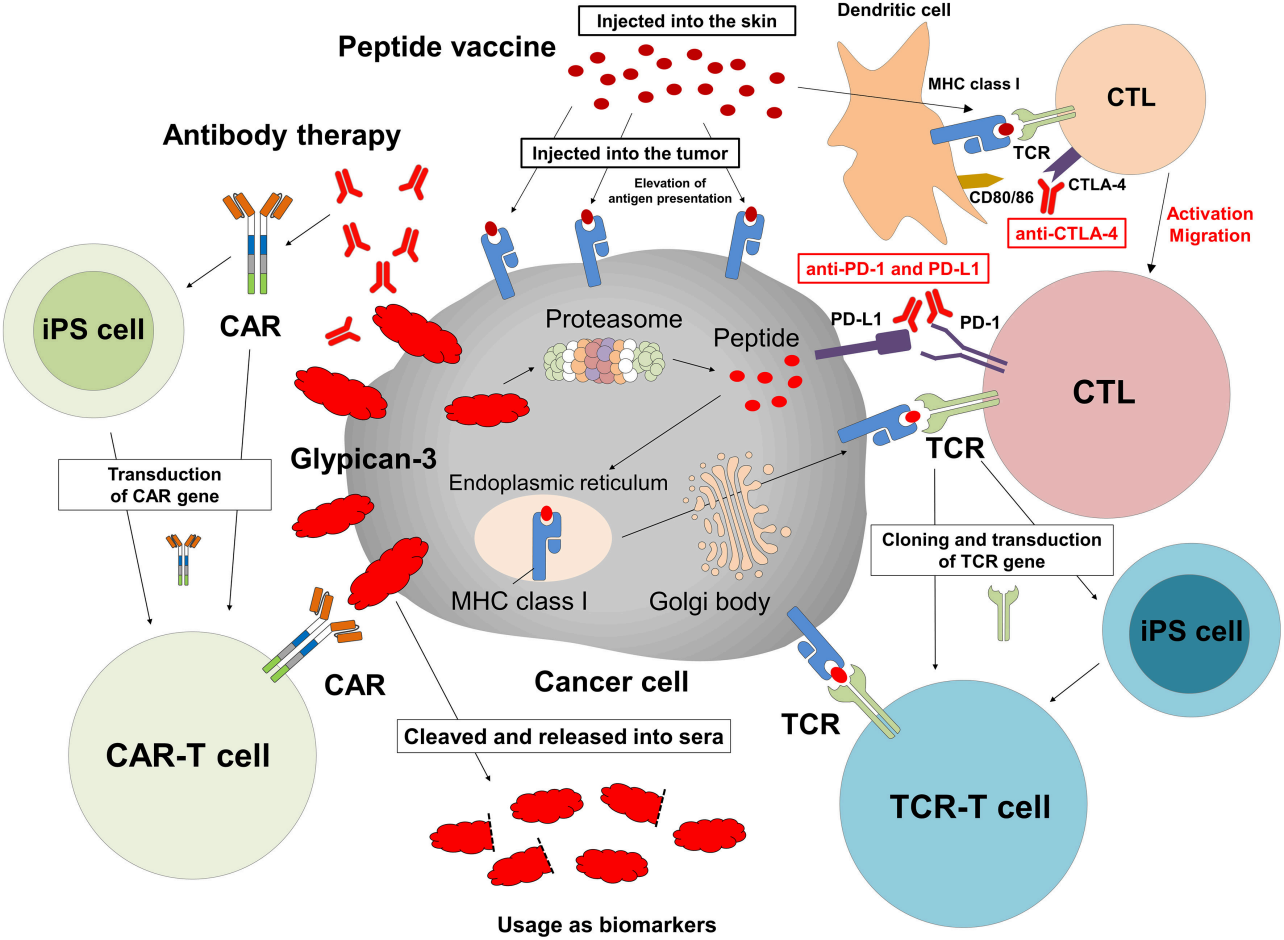
Cancer immunotherapy targeting GPC3. Therapeutic approaches that target intracellular GPC3 include GPC3 peptide vaccine and adoptive immunotherapy based on T cells transduced with a suitable TCR. In contrast, antibody therapy and anti-GPC3-CAR-transduced T cell therapy target membrane-bound GPC3. We are currently developing such T cells and CAR-T cells from iPS cells. In addition, intratumoral vaccination and combination with immune checkpoint inhibitors might enhance effects of these treatments, and serum GPC3 could be a biomarker.

**Figure 3:**
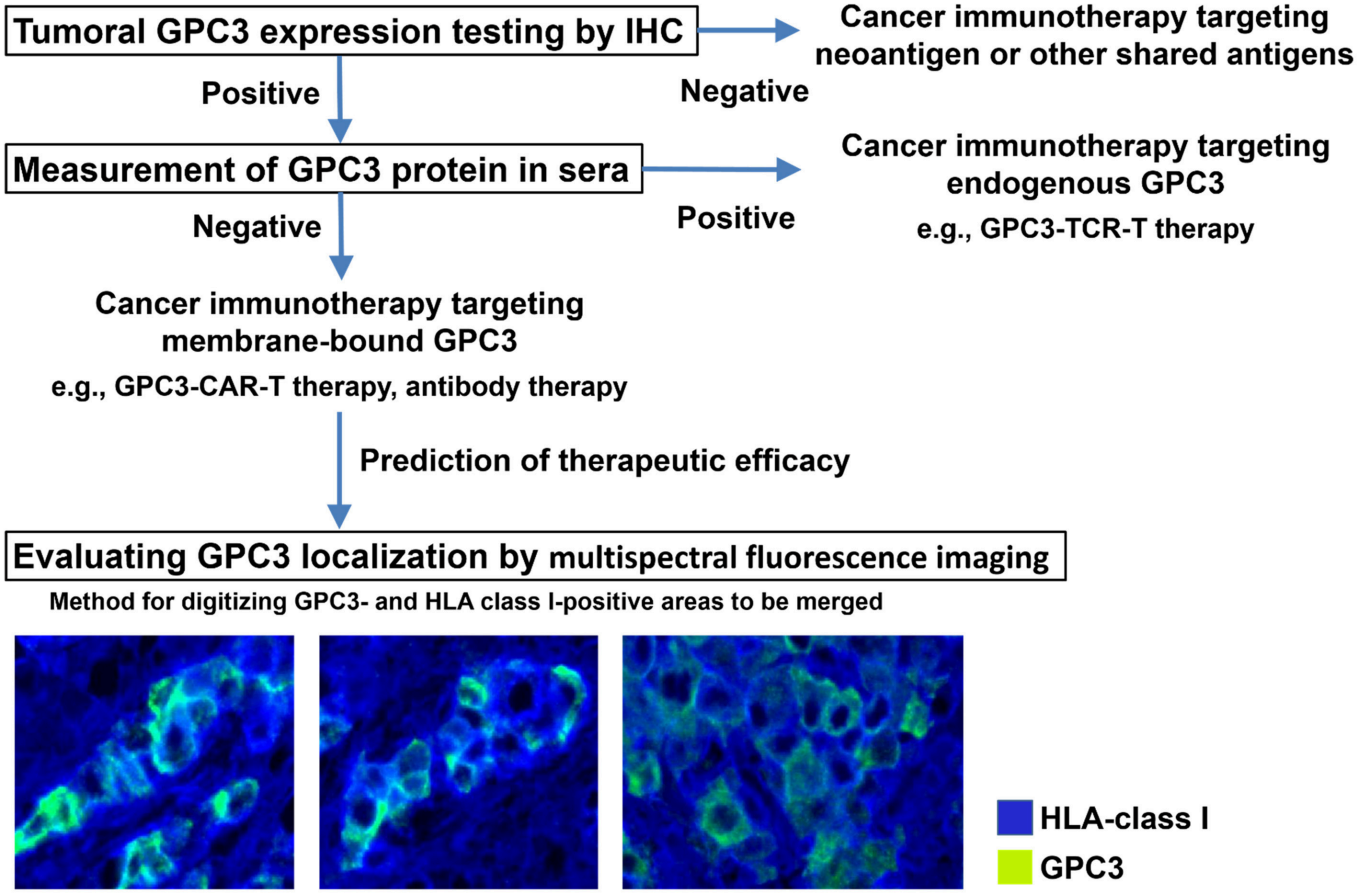
Appropriate treatments targeting GPC3. Analyzing tumor GPC3 expression in combination with serum GPC3 level can reveal the most effective therapeutic strategy. To determine whether GPC3 is predominantly expressed at the cell membrane—which is thought to influence the efficacy of therapies targeting membrane-bound GPC3—we are developing a method of digitizing GPC3- and HLA class I-positive areas to be merged using a multispectral fluorescence imaging system.

We are in the process of developing various treatments and diagnostic methods targeting GPC3 (Figure 2) (65). The subcellular localization of GPC3 and its presence of serum are expected to affect the effects of each treatment approach. Soluble full-length GPC3 protein could block anti-GPC3 antibody and CAR-T cells, thereby reducing cytotoxicity and leading to unexpected side effects. Based on these considerations, serum GPC3 could serve as a biomarker for evaluating treatment effect or be used to assess the eligibility of patients for antibody or GPC3-CAR-T therapy. In addition, the clinical trial for GC33 revealed a correlation between the localization of GPC3 and clinical effects (36). We have also described multiple distinct GPC3 expression patterns (Figure 1C). Therefore, it is important to analyze the localization of GPC3 and not only its presence or absence. To this end we are currently developing a method for investigating the co-expression of GPC3 and class I HLA—a cell membrane-associated molecule—by multiple immunolabeling that can be used as a companion diagnostic tool (Figure 3).

## Future Perspectives

GPC3 has unprecedented cancer specificity and is being studied as a target for cancer immunotherapy worldwide. However, there remain many open questions regarding its natural history, function, and dynamics. Clarifying these aspects of GPC3 is necessary for the development of more effective treatments. Since Boon et al. first identified a cancer-specific antigen for melanoma in 1991, many studies have been undertaken to search for new cancer antigens (66). We identified GPC3 as a tumor-associated antigen and have developed GPC3 peptide vaccine as cancer immunotherapy. Based on findings from our clinical trials, we are now developing the next generation of GPC3-targeting therapeutic approaches such as CAR-T and TCR-engineered T cell therapy. On the other hand, neoantigen is gaining global attention, with significant advances in the establishment of immunotherapies targeting these molecules. We are also developing personalized cancer immunotherapies such as cancer vaccines using peptides derived from neoantigens or individualized adoptive T cell therapies using next-generation sequencers. We look forward to advances in research on neoantigens or shared antigens such as GPC3 that can demonstrate which of these can best serve as immunotherapeutic targets.

## Author Contributions

All authors listed have made a substantial, direct and intellectual contribution to the work, and approved it for publication.

### Conflict of Interest Statement

TN, TS, and TY are supported by fundamental research funding from Takeda Pharmaceutical Co., Ltd. and BrightPath Biotherapeutics Co., Ltd. TN and TS are supported by fundamental research funding from Ono Pharmaceutical Co., Ltd. TN is supported by fundamental research funding from Noile-Immune Biotech Inc. and Sysmex Co., Ltd. The remaining authors declare that the research was conducted in the absence of any commercial or financial relationships that could be construed as a potential conflict of interest.
